# Crystal structure of 1-anilino-5-methyl-1*H*-1,2,3-triazole-4-carb­oxy­lic acid monohydrate

**DOI:** 10.1107/S2056989019005711

**Published:** 2019-05-03

**Authors:** Olívia B. O. Moreira, Maria Clara R. Freitas, Karynne C. Souza, Alessandro K. Jordão, Jackson A. L. C. Resende

**Affiliations:** a Graduate Program in Chemistry, Department of Chemistry, Universidade Federal de Juiz de Fora, Rua José Lourenço Kelmer, s/n, Juiz de Fora - MG, CEP 36036-330, Brazil; b Instituto de Química, Universidade Federal Rural do Rio de Janeiro, BR-465, Km 7, CEP 23.890-000, Seropédica, RJ, Brazil; c Instituto de Ciências Exatas e da Terra, Campus Universitário do Araguaia, Universidade Federal do Mato Grosso, Avenida Universitária, 3500, Pontal do Araguaia - MT, CEP 78698-000, Brazil; d Departamento de Farmácia, Universidade Federal do Rio Grande do Norte, R. Gen. Gustavo Cordeiro de Faria, S/N, Natal - RN, CEP 59012-570, Brazil

**Keywords:** crystal structure, triazole compounds, crystal packing, hydrogen bonding, Hirshfeld surface analysis.

## Abstract

In the mol­ecular structure of the title compound, C_10_H_10_N_4_O_2_·H_2_O, the angle between the triazole and arene rings is 87.39 (5)°. The water of crystallization connects the mol­ecules in the crystal packing. The crystal structure exhibits N—H⋯O, O—H⋯O and O—H⋯N inter­actions, resulting in the formation of a three-dimensional framework. The inter­molecular inter­actions were identified and qu­anti­fied using Hirshfeld surface analysis.

## Abstract 2

The water mol­ecule connects the mol­ecules in the crystal packing. The crystal structure exhibits N—H⋯O, O—H⋯O and O—H⋯N inter­actions, resulting in the formation of a three-dimensional framework.

## Chemical context   

Triazoles are a class of compounds that have aroused chemical inter­est because of their wide range of applications, including their biological relevance and the development of new materials. Triazoles have potent anti­fungal activity, being an important class of drugs (Peyton *et al.*, 2015 ▸). Their anti­tubercular (Zhang *et al.*, 2017 ▸), anti­cancer (Teixeira *et al.*, 2019 ▸), anti­microbial (Yadav *et al.*, 2018 ▸) and anti­viral (Jordão *et al.*, 2009 ▸) activities have also been evaluated. This class of compounds has also aroused inter­est in materials chemistry, mainly in the development of systems with uptake capacity for both CO_2_ and H_2_ (Mukherjee *et al.*, 2019 ▸).
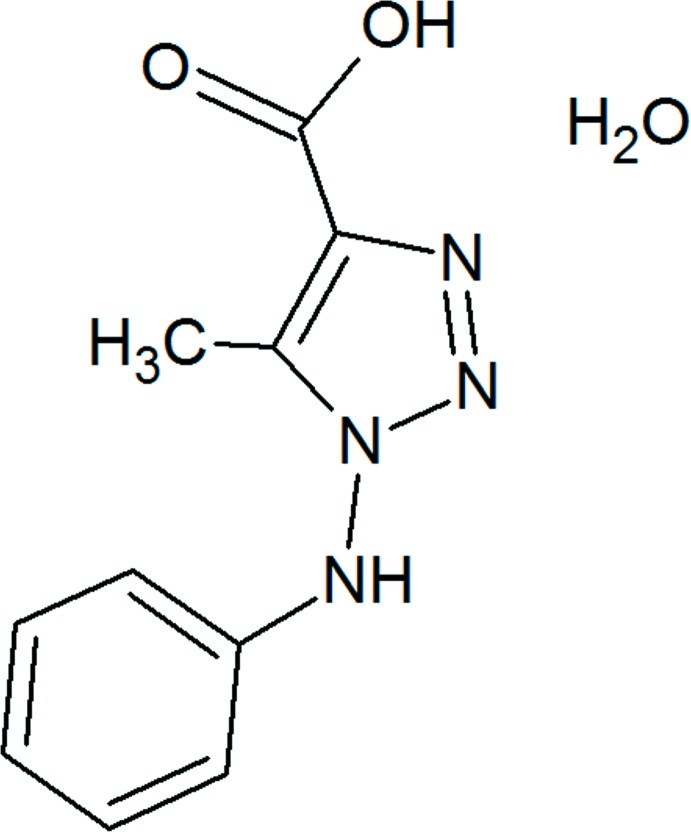


## Structural commentary   

**Figure 1:**
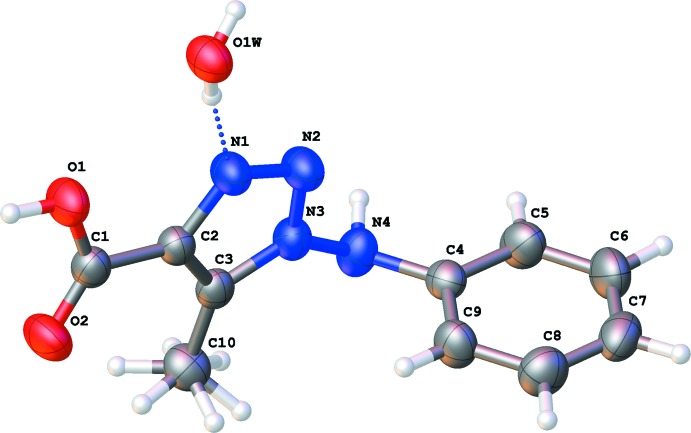
The mol­ecular structure of the title compound with anisotropic atomic displacement ellipsoids shown at the 50% probability level.

The title mol­ecule (Fig. 1 ▸) is formed by planar aniline and triazolic rings, which subtend a dihedral angle of 87.41 (5)°. Atoms O1 and O2 are located 0.237 (2) and 0.208 (2) Å, respectively, outside the plane of the triazole ring. The methyl group exhibits occupational disorder of the hydrogen atoms.

## Supra­molecular features   

**Figure 2:**
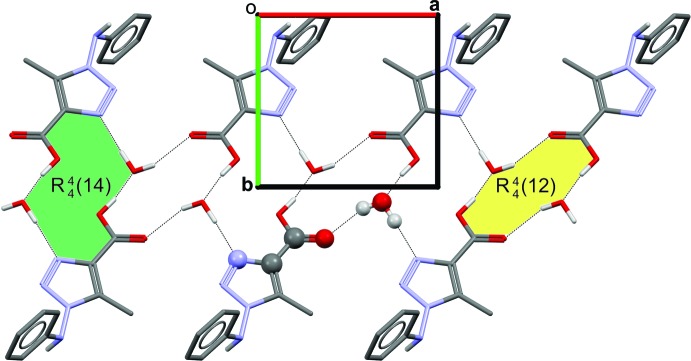
A partial packing diagram showing the hydrogen-bond network along the *a* axis and the 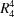
(12) (green) and 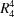
(14) (yellow) motifs. All hydrogen atoms bonded to carbon are omitted for clarity.

**Figure 3:**
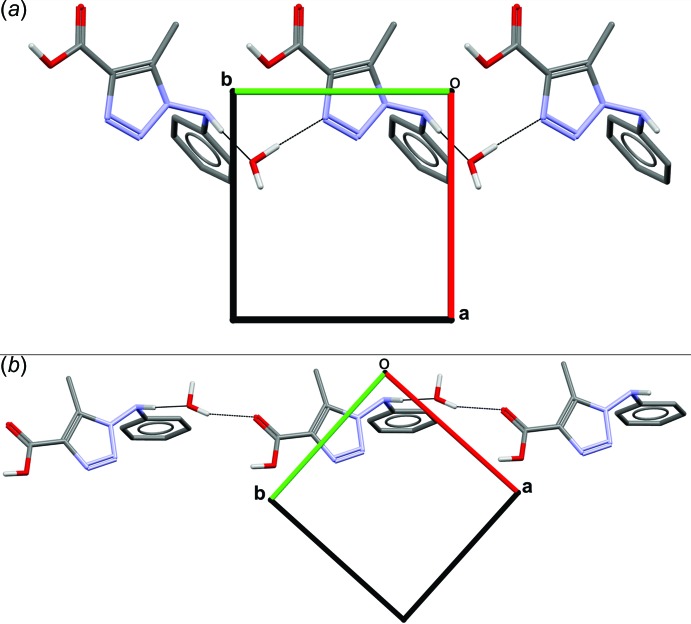
Views along the *c* axis showing the layers consolidated by the hydrogen-bond network: (*a*) a 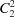
(7) chain along the *b*-axis direction and (*b*) a 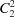
(9) chain along [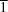
10]. All hydrogen atoms bonded to carbon are omitted for clarity.

The crystal packing is stabilized by N—H⋯O, O—H⋯N and O—H⋯O hydrogen bonds between the water mol­ecule and the organic mol­ecule. The supra­molecular arrangement is formed by four hydrogen bonds (Table 1 ▸): (*A*) N4—H4⋯O1*W* ^ii^, (*B*) O1—H1⋯O1*W* ^i^, (*C*) O1*W*—H1*WA*⋯O2^iii^ and (*D*) O1*W*—H1*WB*⋯N1. Separately, these hydrogen bonds do not form nets in the structure. However, when combined, they generate inter­esting supra­molecular systems. The combination of the (*A*:*B*), (*B*:*C*) and (*B*:*D*) inter­actions result in inter­molecular rings with 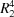
(18), 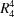
(12) and 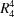
(14) motifs, respectively. Representations of the 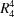
(12) and 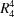
(14) motifs are illustrated in Fig. 2 ▸). A 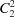
(9) motif is observed along [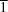
10] (*A*:*C* inter­actions) (Fig. 3*a* ▸), a 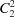
(7) motif along [010] (*A*:*D* inter­actions) (Fig. 3*b* ▸) and a 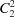
(7) motif along [100] (*C*:*D* inter­actions).

## Hirshfeld surface analysis   

**Figure 4:**
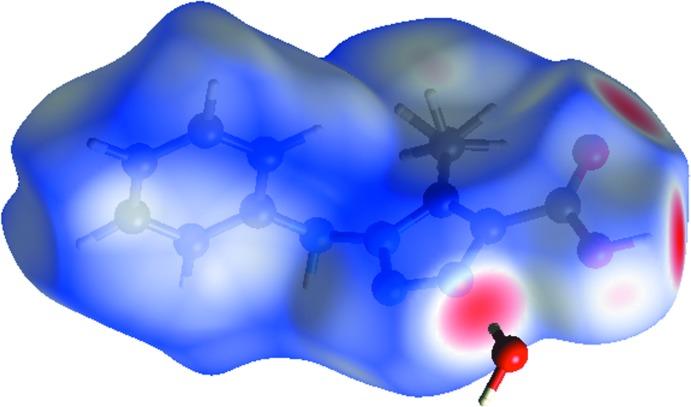
Hirshfeld surface mapped with *d*_norm_.

**Figure 5:**
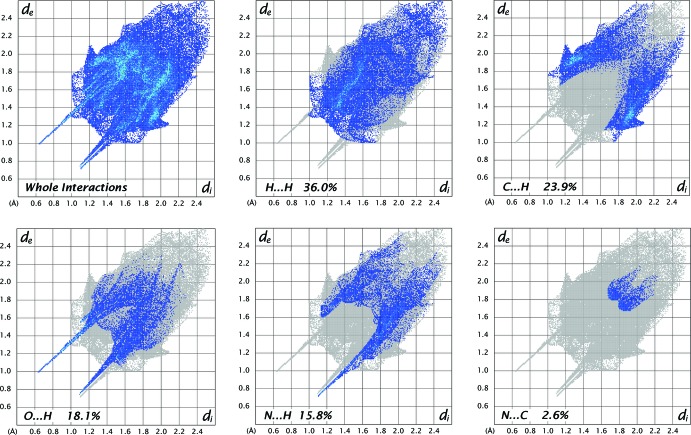
The fingerprint plots for the title compound.

For an unequivocal description of the supra­molecular system, Hirshfeld Surface (HS) analysis was performed. The isosurface was plotted for the weight function equal to 0.5. The red areas in Fig. 4 ▸ correspond to short contacts between atoms inside and outside the surface atom, *d*_i_ and *d*_e_. There are three spots on the surface, and in the corresponding fingerprint plot (FPP; Fig. 5 ▸), they are represented as sharp spikes. Chemically, they correspond to classical hydrogen bonds. Two of these involve inter­actions between the carboxyl group and the water mol­ecule while the third is the inter­action between *N*-triazole and the water mol­ecule. These hydrogen bonds are the shortest contacts, assigned in the FPP as O⋯H and N⋯H. The N⋯H inter­action contributes 15.8% to the HS, while the O⋯H inter­action corresponds to 18.1%. The majority of the inter­actions are H⋯H, being equal to 36.0%.

## Database survey   

A research of the Cambridge Structural Database (CSD version 5.40, update of November 2018; Groom *et al.*, 2016 ▸) for *N*-phenyl-1*H*-1,2,3-triazol-1-amine derivatives gave 18 hits for structures that include atomic coordinates. These results include alcohols, esters and a carbohydrazide. The mol­ecular structures of these compounds show dihedral angles between the triazole and aniline rings in the range 76 to 89°. These values are affected by the hydrogen bonds in the crystal packing. In addition, in studies of halogenated phenyl derivatives, differences in C—H⋯π inter­actions were shown to result in changes in the crystal packing (Jordão *et al.*, 2012 ▸).

## Vibrational spectrum   

**Figure 6:**
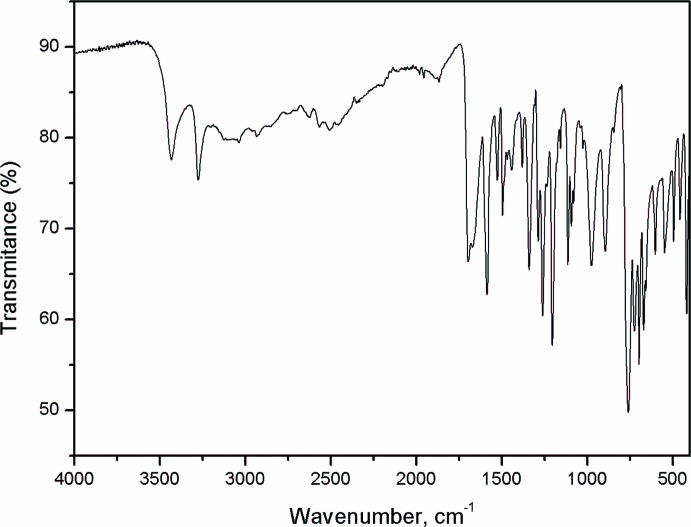
IR spectrum of the title compound.

Fig. 6 ▸ shows the IR spectrum measured in ATR mode (ν_max_, cm^−1^) which exhibits the following characteristic bands: 3205 (N—H stretching); 2984 (methyl C—H stretching); 1725 (C=O stretching); 1600 (>C=N stretching); 1496 (aromatic C=C stretching); 1348 (C—N stretching of triazole); 1208 (C—O stretching) for the esther and 3431 (OH stretching); 3268 (N—H stretching); 1695 (C=O stretching); 1589 (>C=N stretching); 1496 (aromatic C=C stretching); 1381 (C—N stretching of triazole); 1259 (C—O stretching) for the acid.

## Synthesis and crystallization   

The title compound was synthesized by the alkaline hydrolysis of 5-methyl-1-(phenyl­amino)-1*H*-[1,2,3]-triazole-4-carb­oxy­lic acid ethyl ester (Jordão *et al.*, 2009 ▸), **1**. 3.6 mmol of **1** were dissolved in 30.0 ml of a sodium hydroxide solution (0.1 mol L^−1^) (NaOH, VETEC). This mixture was refluxed at 373 K for about 48 h. The product was neutralized using dilute hydro­chloric acid (HCl, VETEC), filtered and dried *in vacuo*. The title compound was dissolved in methanol and kept at room temperature. After a few days, colourless block-shaped crystals, suitable for X-ray analysis, were obtained by slow evaporation (yield 83%).

^1^H NMR (500 MHz, C_2_D_6_OS): 10.218 (1H, *s*), 9.887 (1H, *s*), 7.215 (2H, *m*), 6.872 (1H, *m*), 6.390(2H, *d*, *J* = 3Hz), 3.295 (1H, *s*).

## Refinement   

Crystal data, data collection and structure refinement details are summarized in Table 2 ▸. All H atoms were located in a difference-Fourier map and freely refined except for hydrogen atoms bound to C10 which are disordered (occupancy 0.5) and were refined using a riding model with C—H = 0.96 Å and *U*_iso_(H) = 1.5*U*_eq_(C).

## Supplementary Material

Crystal structure: contains datablock(s) I. DOI: 10.1107/S2056989019005711/ex2020sup1.cif

Structure factors: contains datablock(s) I. DOI: 10.1107/S2056989019005711/ex2020Isup2.hkl

Supporting information file. DOI: 10.1107/S2056989019005711/ex2020Isup3.cml

CCDC reference: 1912546

Additional supporting information:  crystallographic information; 3D view; checkCIF report

## Figures and Tables

**Table 1:** Hydrogen-bond geometry (Å, °)

*D*—H⋯*A*	*D*—H	H⋯*A*	*D*⋯*A*	*D*—H⋯*A*
O1—H1⋯O1*W* ^i^	0.94 (2)	1.68 (3)	2.6030 (19)	167 (2)
N4—H4⋯O1*W* ^ii^	0.91 (2)	2.22 (2)	3.111 (2)	169.2 (19)
O1*W*—H1*WA*⋯O2^iii^	0.82 (3)	1.99 (3)	2.773 (2)	159 (2)
O1*W*—H1*WB*⋯N1	0.92 (3)	1.88 (3)	2.800 (2)	172 (2)

Symmetry codes: (i) 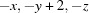; (ii) 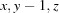; (iii) 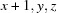.

**Table 2:** Experimental details

Crystal data	
Chemical formula	C_10_H_10_N_4_O_2_·H_2_O
*M*_r_	236.24
Crystal system, space group	Monoclinic, *P*2_1_/*c*
Temperature (K)	298
*a*, *b*, *c* (Å)	7.2288 (14), 6.8265 (14), 23.922 (5)
β (°)	98.69 (3)
*V* (Å^3^)	1167.0 (4)
*Z*	4
Radiation type	Mo *K*α
μ (mm^−1^)	0.10
Crystal size (mm)	0.24 × 0.20 × 0.06

Data collection	
Diffractometer	Bruker KappaCCD
Absorption correction	Multi-scan (*SADABS*; Bruker, 2008 ▸)
*T*_min_, *T*_max_	0.701, 0.745
No. of measured, independent and observed [*I* > 2σ(*I*)] reflections	11378, 2207, 1559
*R*_int_	0.042
(sin θ/λ)_max_ (Å^−1^)	0.609

Refinement	
*R*[*F* ^2^ > 2σ(*F* ^2^)], *wR*(*F* ^2^), *S*	0.041, 0.100, 1.04
No. of reflections	2207
No. of parameters	191
H-atom treatment	H atoms treated by a mixture of independent and constrained refinement
Δρ_max_, Δρ_min_ (e Å^−3^)	0.18, −0.15

Computer programs: *COLLECT* (Bruker, 2004 ▸), *DIRAX/LSQ* (Duisenberg, 1992 ▸), *EVALCCD* (Duisenberg *et al.*, 2003 ▸), *SHELXT* (Sheldrick, 2015*a* ▸), *SHELXL2016* (Sheldrick, 2015*b* ▸), *OLEX2* (Dolomanov *et al.*, 2009 ▸) and *Mercury* (Macrae *et al.*, 2008 ▸).

## supplementary crystallographic information

### Crystal data

**Table:**

C_10_H_10_N_4_O_2_·H_2_O	*F*(000) = 496
*M**_r_* = 236.24	*D*_x_ = 1.345 Mg m^−^^3^
Monoclinic, *P*2_1_/*c*	Mo *K*α radiation, λ = 0.71073 Å
*a* = 7.2288 (14) Å	Cell parameters from 11378 reflections
*b* = 6.8265 (14) Å	θ = 3.1–25.2°
*c* = 23.922 (5) Å	µ = 0.10 mm^−^^1^
β = 98.69 (3)°	*T* = 298 K
*V* = 1167.0 (4) Å^3^	Block, colourless
*Z* = 4	0.24 × 0.20 × 0.06 mm

### Data collection

**Table:**

Bruker KappaCCD diffractometer	2207 independent reflections
Horizonally mounted graphite crystal monochromator	1559 reflections with *I* > 2σ(*I*)
Detector resolution: 9 pixels mm^-1^	*R*_int_ = 0.042
CCD scans	θ_max_ = 25.7°, θ_min_ = 3.1°
Absorption correction: multi-scan (SADABS; Bruker, 2008)	*h* = −8→8
*T*_min_ = 0.701, *T*_max_ = 0.745	*k* = −8→7
11378 measured reflections	*l* = −28→29

### Refinement

**Table:**

Refinement on *F*^2^	Hydrogen site location: mixed
Least-squares matrix: full	H atoms treated by a mixture of independent and constrained refinement
*R*[*F*^2^ > 2σ(*F*^2^)] = 0.041	*w* = 1/[σ^2^(*F*_o_^2^) + (0.0451*P*)^2^ + 0.2317*P*] where *P* = (*F*_o_^2^ + 2*F*_c_^2^)/3
*wR*(*F*^2^) = 0.100	(Δ/σ)_max_ < 0.001
*S* = 1.04	Δρ_max_ = 0.18 e Å^−^^3^
2207 reflections	Δρ_min_ = −0.15 e Å^−^^3^
191 parameters	Extinction correction: SHELXL2016 (Sheldrick, 2015b), Fc^*^=kFc[1+0.001xFc^2^λ^3^/sin(2θ)]^-1/4^
0 restraints	Extinction coefficient: 0.016 (3)

### Special details

**Table:**

Geometry. All esds (except the esd in the dihedral angle between two l.s. planes) are estimated using the full covariance matrix. The cell esds are taken into account individually in the estimation of esds in distances, angles and torsion angles; correlations between esds in cell parameters are only used when they are defined by crystal symmetry. An approximate (isotropic) treatment of cell esds is used for estimating esds involving l.s. planes.

### Fractional atomic coordinates and isotropic or equivalent isotropic displacement parameters (Å^2^)

**Table:**

	*x*	*y*	*z*	*U*_iso_*/*U*_eq_	Occ. (<1)
N1	0.1134 (2)	0.5773 (2)	0.07579 (6)	0.0470 (4)	
N2	0.1952 (2)	0.4253 (2)	0.10201 (7)	0.0490 (4)	
N3	0.0553 (2)	0.3016 (2)	0.11042 (6)	0.0408 (4)	
N4	0.0956 (2)	0.1211 (2)	0.13675 (6)	0.0456 (4)	
O1	−0.11452 (18)	0.8386 (2)	0.01606 (6)	0.0548 (4)	
O2	−0.37050 (18)	0.6983 (2)	0.03987 (6)	0.0626 (4)	
C1	−0.2030 (3)	0.7017 (3)	0.04017 (7)	0.0416 (4)	
C2	−0.0763 (2)	0.5512 (3)	0.06831 (7)	0.0379 (4)	
C3	−0.1165 (2)	0.3727 (2)	0.09034 (7)	0.0375 (4)	
C4	0.1777 (2)	0.1351 (2)	0.19481 (7)	0.0387 (4)	
C5	0.3163 (3)	0.0044 (3)	0.21538 (9)	0.0536 (5)	
C6	0.3937 (3)	0.0088 (3)	0.27196 (9)	0.0608 (6)	
C7	0.3320 (3)	0.1427 (3)	0.30781 (9)	0.0552 (6)	
C8	0.1928 (3)	0.2721 (3)	0.28731 (9)	0.0575 (5)	
C9	0.1139 (3)	0.2681 (3)	0.23076 (8)	0.0502 (5)	
C10	−0.2922 (3)	0.2638 (3)	0.09330 (8)	0.0507 (5)	
H10A	−0.397146	0.341238	0.076648	0.076*	0.5
H10B	−0.290788	0.142584	0.073046	0.076*	0.5
H10C	−0.302575	0.237247	0.132110	0.076*	0.5
H10D	−0.263193	0.139474	0.111221	0.076*	0.5
H10E	−0.369552	0.338129	0.114823	0.076*	0.5
H10F	−0.357764	0.243466	0.055760	0.076*	0.5
O1W	0.3100 (2)	0.9111 (2)	0.05054 (6)	0.0509 (4)	
H1	−0.199 (3)	0.926 (4)	−0.0049 (10)	0.081 (7)*	
H9	0.022 (3)	0.353 (3)	0.2172 (8)	0.058 (6)*	
H5	0.359 (3)	−0.091 (3)	0.1907 (9)	0.067 (6)*	
H6	0.495 (3)	−0.078 (3)	0.2851 (9)	0.076 (7)*	
H7	0.388 (3)	0.149 (3)	0.3456 (10)	0.067 (6)*	
H8	0.139 (3)	0.367 (4)	0.3125 (10)	0.083 (7)*	
H4	0.170 (3)	0.056 (3)	0.1157 (9)	0.063 (6)*	
H1WA	0.415 (4)	0.876 (4)	0.0455 (10)	0.074 (8)*	
H1WB	0.241 (4)	0.800 (4)	0.0552 (10)	0.089 (8)*	

### Atomic displacement parameters (Å^2^)

**Table:**

	*U*^11^	*U*^22^	*U*^33^	*U*^12^	*U*^13^	*U*^23^
N1	0.0395 (9)	0.0483 (9)	0.0530 (9)	0.0066 (7)	0.0060 (7)	0.0148 (8)
N2	0.0410 (9)	0.0486 (9)	0.0570 (10)	0.0059 (8)	0.0067 (7)	0.0150 (8)
N3	0.0423 (9)	0.0390 (8)	0.0404 (8)	0.0054 (7)	0.0043 (6)	0.0064 (7)
N4	0.0573 (10)	0.0355 (9)	0.0431 (9)	0.0114 (7)	0.0046 (7)	0.0046 (7)
O1	0.0467 (8)	0.0527 (8)	0.0641 (9)	0.0101 (7)	0.0057 (7)	0.0233 (7)
O2	0.0394 (8)	0.0704 (10)	0.0785 (10)	0.0141 (7)	0.0102 (7)	0.0187 (8)
C1	0.0426 (11)	0.0450 (11)	0.0374 (9)	0.0082 (9)	0.0068 (8)	0.0011 (8)
C2	0.0361 (10)	0.0419 (10)	0.0355 (9)	0.0054 (8)	0.0051 (7)	0.0020 (8)
C3	0.0407 (10)	0.0403 (10)	0.0313 (9)	0.0053 (8)	0.0053 (7)	−0.0019 (8)
C4	0.0390 (10)	0.0348 (9)	0.0428 (10)	0.0029 (8)	0.0080 (8)	0.0070 (8)
C5	0.0580 (13)	0.0519 (12)	0.0522 (12)	0.0201 (10)	0.0129 (10)	0.0078 (10)
C6	0.0533 (13)	0.0678 (15)	0.0594 (14)	0.0172 (11)	0.0027 (10)	0.0192 (12)
C7	0.0569 (13)	0.0638 (14)	0.0433 (11)	−0.0076 (11)	0.0022 (10)	0.0109 (11)
C8	0.0658 (14)	0.0566 (13)	0.0506 (12)	0.0041 (11)	0.0107 (10)	−0.0039 (11)
C9	0.0498 (12)	0.0487 (12)	0.0512 (12)	0.0146 (10)	0.0045 (9)	0.0020 (10)
C10	0.0478 (11)	0.0478 (11)	0.0557 (12)	−0.0048 (9)	0.0053 (9)	−0.0045 (9)
O1W	0.0430 (9)	0.0445 (8)	0.0649 (9)	0.0073 (7)	0.0074 (7)	0.0108 (7)

### Geometric parameters (Å, º)

**Table:**

N1—N2	1.307 (2)	C5—H5	0.96 (2)
N1—C2	1.367 (2)	C6—C7	1.373 (3)
N2—N3	1.356 (2)	C6—H6	0.96 (2)
N3—C3	1.352 (2)	C7—C8	1.372 (3)
N3—N4	1.394 (2)	C7—H7	0.93 (2)
N4—C4	1.429 (2)	C8—C9	1.387 (3)
N4—H4	0.91 (2)	C8—H8	1.00 (2)
O1—C1	1.314 (2)	C9—H9	0.90 (2)
O1—H1	0.94 (2)	C10—H10A	0.9600
O2—C1	1.210 (2)	C10—H10B	0.9600
C1—C2	1.470 (2)	C10—H10C	0.9600
C2—C3	1.376 (2)	C10—H10D	0.9600
C3—C10	1.482 (2)	C10—H10E	0.9600
C4—C5	1.376 (3)	C10—H10F	0.9600
C4—C9	1.377 (3)	O1W—H1WA	0.82 (3)
C5—C6	1.385 (3)	O1W—H1WB	0.92 (3)
			
N2—N1—C2	109.36 (14)	C6—C7—H7	120.2 (13)
N1—N2—N3	105.80 (14)	C7—C8—C9	120.5 (2)
C3—N3—N2	112.91 (14)	C7—C8—H8	122.1 (13)
C3—N3—N4	126.55 (15)	C9—C8—H8	117.3 (14)
N2—N3—N4	120.53 (14)	C4—C9—C8	119.66 (19)
N3—N4—C4	114.07 (14)	C4—C9—H9	119.7 (13)
N3—N4—H4	106.5 (13)	C8—C9—H9	120.6 (13)
C4—N4—H4	112.3 (14)	C3—C10—H10A	109.5
C1—O1—H1	111.4 (14)	C3—C10—H10B	109.5
O2—C1—O1	124.32 (17)	H10A—C10—H10B	109.5
O2—C1—C2	122.91 (17)	C3—C10—H10C	109.5
O1—C1—C2	112.77 (15)	H10A—C10—H10C	109.5
N1—C2—C3	109.33 (15)	H10B—C10—H10C	109.5
N1—C2—C1	120.80 (15)	C3—C10—H10D	109.5
C3—C2—C1	129.87 (16)	H10A—C10—H10D	141.1
N3—C3—C2	102.58 (14)	H10B—C10—H10D	56.3
N3—C3—C10	123.43 (16)	H10C—C10—H10D	56.3
C2—C3—C10	133.97 (16)	C3—C10—H10E	109.5
C5—C4—C9	119.92 (18)	H10A—C10—H10E	56.3
C5—C4—N4	118.51 (16)	H10B—C10—H10E	141.1
C9—C4—N4	121.46 (16)	H10C—C10—H10E	56.3
C4—C5—C6	120.0 (2)	H10D—C10—H10E	109.5
C4—C5—H5	120.3 (13)	C3—C10—H10F	109.5
C6—C5—H5	119.7 (13)	H10A—C10—H10F	56.3
C7—C6—C5	120.3 (2)	H10B—C10—H10F	56.3
C7—C6—H6	120.8 (13)	H10C—C10—H10F	141.1
C5—C6—H6	118.8 (13)	H10D—C10—H10F	109.5
C8—C7—C6	119.7 (2)	H10E—C10—H10F	109.5
C8—C7—H7	120.1 (13)	H1WA—O1W—H1WB	108 (2)
			
C2—N1—N2—N3	0.73 (19)	N1—C2—C3—N3	0.32 (18)
N1—N2—N3—C3	−0.55 (19)	C1—C2—C3—N3	−179.89 (16)
N1—N2—N3—N4	178.75 (14)	N1—C2—C3—C10	−178.06 (18)
C3—N3—N4—C4	−114.38 (18)	C1—C2—C3—C10	1.7 (3)
N2—N3—N4—C4	66.4 (2)	N3—N4—C4—C5	−141.88 (17)
N2—N1—C2—C3	−0.69 (19)	N3—N4—C4—C9	41.9 (2)
N2—N1—C2—C1	179.51 (15)	C9—C4—C5—C6	−1.1 (3)
O2—C1—C2—N1	−168.96 (17)	N4—C4—C5—C6	−177.43 (18)
O1—C1—C2—N1	10.7 (2)	C4—C5—C6—C7	0.3 (3)
O2—C1—C2—C3	11.3 (3)	C5—C6—C7—C8	0.1 (3)
O1—C1—C2—C3	−169.10 (17)	C6—C7—C8—C9	0.2 (3)
N2—N3—C3—C2	0.13 (18)	C5—C4—C9—C8	1.4 (3)
N4—N3—C3—C2	−179.11 (15)	N4—C4—C9—C8	177.62 (18)
N2—N3—C3—C10	178.74 (15)	C7—C8—C9—C4	−1.0 (3)
N4—N3—C3—C10	−0.5 (3)		

### Hydrogen-bond geometry (Å, º)

**Table:**

*D*—H···*A*	*D*—H	H···*A*	*D*···*A*	*D*—H···*A*
O1—H1···O1*W*^i^	0.94 (2)	1.68 (3)	2.6030 (19)	167 (2)
N4—H4···O1*W*^ii^	0.91 (2)	2.22 (2)	3.111 (2)	169.2 (19)
O1*W*—H1*WA*···O2^iii^	0.82 (3)	1.99 (3)	2.773 (2)	159 (2)
O1*W*—H1*WB*···N1	0.92 (3)	1.88 (3)	2.800 (2)	172 (2)

Symmetry codes: (i) −*x*, −*y*+2, −*z*; (ii) *x*, *y*−1, *z*; (iii) *x*+1, *y*, *z*.
